# Using crop intercepted solar radiation and vegetation index to estimate dry matter yield of Choy Sum

**DOI:** 10.3389/fpls.2023.1208404

**Published:** 2023-09-18

**Authors:** Yiyin He, Zhao Wang, Sashuang Sun, Lijun Zhu, Yu Li, Xiaoxiao Wang, Jiang Shi, Si Chen, Dunchang Qi, Junxiang Peng, Zhenjiang Zhou

**Affiliations:** ^1^College of Biosystems Engineering and Food Science, Zhejiang University, Hangzhou, China; ^2^Huanan Industrial Technology Research Institute of Zhejiang University, Guangzhou, China; ^3^Agricultural Experiment Station, Zhejiang University, Hangzhou, China; ^4^Hangzhou Academy of Agricultural Sciences, Hangzhou, China; ^5^College of Water Resources and Environmental Engineering, Zhejiang University of Water Resources and Electric Power, Hangzhou, China; ^6^Department of Agricultural Research for Northern Sweden, Swedish University of Agricultural Sciences, Umeå, Sweden

**Keywords:** Choy Sum, canopy coverage, radiation use efficiency, dry matter, N fertilization

## Abstract

An accurate assessment of vegetable yield is essential for agricultural production and management. One approach to estimate yield with remote sensing is via vegetation indices, which are selected in a statistical and empirical approach, rather than a mechanistic way. This study aimed to estimate the dry matter of Choy Sum by both a causality-guided intercepted radiation-based model and a spectral reflectance-based model and compare their performance. Moreover, the effect of nitrogen (N) rates on the radiation use efficiency (*RUE*) of Choy Sum was also evaluated. A 2-year field experiment was conducted with different N rate treatments (0 kg/ha, 25 kg/ha, 50 kg/ha, 100 kg/ha, 150 kg/ha, and 200 kg/ha). At different growth stages, canopy spectra, photosynthetic active radiation, and canopy coverage were measured by RapidScan CS-45, light quantum sensor, and camera, respectively. The results reveal that exponential models best match the connection between dry matter and vegetation indices, with coefficients of determination (*R*^2^) all below 0.80 for normalized difference red edge (NDRE), normalized difference vegetation index (NDVI), red edge ratio vegetation index (RERVI), and ratio vegetation index (RVI). In contrast, accumulated intercepted photosynthetic active radiation (*Aipar*) showed a significant linear correlation with the dry matter of Choy Sum, with root mean square error (*RMSE*) of 9.4 and *R*^2^ values of 0.82, implying that the *Aipar*-based estimation model performed better than that of spectral-based ones. Moreover, the *RUE* of Choy Sum was significantly affected by the N rate, with 100 kg N/ha, 150 kg N/ha, and 200 kg N/ha having the highest *RUE* values. The study demonstrated the potential of *Aipar*-based models for precisely estimating the dry matter yield of vegetable crops and understanding the effect of N application on dry matter accumulation of Choy Sum.

## 1 Introduction

Choy Sum (*Brassica rapa* var. *parachinensis*) is one of the most productive and consumed vegetables in Asia (Kok et al., 1991; Zou et al., 2021). Accurate estimation of its dry matter yield is vital for the assessment of crop performance and decision-making during the growth season. Laboratory analysis of fresh or dry plants is conventionally performed to determine yield, but the process is time- and labor-intensive. Furthermore, plants are typically sampled on limited spots, which may not accurately represent dry matter yield across the complete region. Therefore, a quick and precise method for determining the dry matter yield of Choy Sum is required.

Due to the non-destructive, accurate, and timely access to crop information, the spectral-based remote sensing method has been used extensively in agriculture to estimate the crop agronomy parameters including nitrogen concentration, leaf area index, canopy coverage, and biomass (Mulla, 2013; Pereira et al., 2020). One typical approach is to use statistically selected vegetation indices to estimate crop dry matter of wheat (Erdle et al., 2011; Elsayed et al., 2021), rice (Gnyp et al., 2014), potato (Liu et al., 2022), etc. However, vegetation indices are prone to saturation at dense canopies, resulting in decreased accuracy of the estimation model (Wang et al., 2012; Gnyp et al., 2014). This is mainly due to the fact that spectral penetration at a specific wavelength is constrained on dense canopies, a mechanistic method that takes into account that plant growth is needed to address the problem of saturation. Thus, a method based on canopy intercepted solar radiation and its use efficiency is proposed in this study.

As plant dry matter (DM) is the production of photosynthetic activity, which uses sun radiation, it is determined by accumulated intercepted photosynthetic active radiation (*Aipar*) and the efficiency of using it (Leblon et al., 1991; Andersen et al., 1996). This can be expressed in the form of DM = *RUE* × *Aipar*, where *RUE* is the radiation use efficiency. *RUE* is a paramount parameter for crop growth models. Among all crop management factors, nitrogen (N) application rate can affect crop dry matter via its effects on *Aipar*, *RUE*, or both (Shah et al., 2004; Zhou et al., 2016). *RUE* is primarily controlled by crop net photosynthesis (Monteith, 1994), which in turn has a close dependency on leaf N concentration (Lawlor et al., 1989). Therefore, N fertilization affects *RUE* by influencing the photosynthesis rate (Muchow and Sinclair, 1994). Despite the fact that numerous studies have been conducted on the impact of N on *RUE* or *Aipar* (Chakwizira et al., 2018; Wang et al., 2020; Cafaro La Menza et al., 2022), very few of them have focused on the vegetable crop. Thus, the impact of N rates on the *RUE* of Choy Sum needs to be investigated. In addition, the potential of using the *Aipar*-based method to estimate the dry matter of vegetable crops is still unknown.

Therefore, the purposes of this study were to 1) explore the capacity of *Aipar* to estimate the dry matter yield of Choy Sum and 2) investigate the effect of N rates on the radiation use efficiency of Choy Sum.

## 2 Materials and methods

### 2.1 Field experiments

A 2-year field experiment was carried out from 2021 to 2022 at the Qiyuan farm site (30°100E, 119°480N), Hangzhou City, China. The experimental site has a typical subtropical monsoon climate condition and is characterized by abundant precipitation with mild seasonal temperature variation. The yearly mean annual temperature and total precipitation are 18.7°C and 1,930 mm, respectively. The predominant soil is characterized as loamy, having a pH of 7.9, total N content of 1.45 g/kg, hydrolytic N content of 199 mg/kg, and organic matter of 24.1 g/kg.

In 2021, Choy Sum was transplanted on April 15 and harvested on May 24. In 2022, Choy Sum was transplanted on April 1 and harvested on April 25. The average temperature was 22.9°C and the total precipitation was 147.0 mm in the growth season of 2021, while the average temperature was 18.1°C and the total precipitation was 121.7 mm in the growth season of 2022. Pumpkin and sweet potato were the previous crops in both seasons. Each plot had a size of 5 m × 5 m. The plant density was 0.15 m × 0.15 m and 0.3 m × 0.3 m in 2021 and 2022, respectively. Six treatments were included in each experiment differentiated by varying N rates of 0 kg/ha, 25 kg/ha, 50 kg/ha, 100 kg/ha, 150 kg/ha, and 200 kg/ha (defined as N_0_, N_25_, N_50_, N_100_, N_150_, and N_200_, respectively). The experimental design utilized in the field was a completely randomized block design, which consisted of three replications. Urea was used to apply N fertilizer at transplantation, while 30 kg/ha of phosphorus (P) and 90 kg/ha of potassium (K) were applied simultaneously. The administration of irrigation, pest control, and disease management were conducted in accordance with regional best practices.

### 2.2 Sample collection and data acquisition

A total of 90 and 54 samples were collected in 2021 and 2022, respectively (**Table 1**). Each plot had a sub-plot of 0.5 m × 0.7 m marked for sampling, and plants within the sub-plot area were collected and stored for subsequent analysis. Digital images and spectral measurements were taken prior to destructive sampling. All samples were desiccated in the oven at 105° C for 30 minutes and then at 75° C for 48 hours to measure dry matter, which refers to the dry weight of above-ground plant material per unit area. Subsequently, plant N concentration (*PNC*, %) of dried samples was determined by the Kjeldahl method (KDN-B, Shanghai Xinjia Electronic, Co., Ltd., Shanghai, China).

**Table 1:** Field experimental design in 2021 and 2022.

Year	Transplanting date	Harvest date	Replicates	N rates (kg N/ha)	Sampling time (days after transplanting)
**2021**	April 15	May 24	3	0, 25, 50, 100,150, 200	15–21–24–27–31
**2022**	April 1	April 25	3	0, 25, 50, 100,150, 200	15–20–25

A hand-held field spectrometer (RapidScan CS-45; Holland Scientific, Lincoln, NE, USA) was used to measure canopy spectral reflectance at 670 nm (defined as Red), 730 nm (defined as RedEdge), and 780 nm (defined as near infrared (NIR)) wavelength and provides normalized difference red edge (NDRE) and normalized difference vegetation index (NDVI) (Aranguren et al., 2019). For each plot, a representative row was selected for spectral data measurement. The instrument was maintained approximately 1 m above the crop canopy during the scanning procedure. The four most commonly used vegetation indices named NDRE, NDVI, red edge ratio vegetation index (RERVI) (Erdle et al., 2011), and ratio vegetation index (RVI) (Zhou et al., 2017) were calculated as follows.


(1) NDRE=(NIR − RedEdge) / (NIR + RedEdge),

(2) NDVI=(NIR − Red) / (NIR + Red),

(3) RERVI=NIR / RedEdge, 

(4) RVI=NIR / Red.

A digital camera (D5600, Nikon, Tokyo, Japan) was used to capture crop images, which were held above the crop canopy at a distance of 1 m. Images were converted to HSI three-color space (hue, saturation, and intensity channels) using Matlab (The MathWorks, Inc., Natick, MA, USA) programming. The threshold of H values was used to generate binary pictures. Those pixels with H values between 0.2 and 0.4 were given the value 1 (green vegetation), while other pixels without vegetation were put to 0 (soil background). Canopy coverage was calculated as the proportion of the number of pixels with a value of 1 to the whole pixel number in the binary image.

### 2.3 Radiation interception measurement by light quantum sensor

Photosynthetic active radiation was measured by a light quantum sensor (model Li-190R, Li-Cor Inc., Lincoln, NE, USA), which was installed near the experimental site. The radiation sensor was connected to a data logger (model CR3000 Series), which was used to record incident photosynthetic active radiation every 10 minutes. Daily intercepted photosynthetic active par (*Ipar*) and accumulated *Ipar* of plant canopy were calculated as follows:


(5) $$Ipar=I_{0}Fipar,$$

(6) Aipar=∑Ipar dt,

where *I*_0_ is photosynthetic active radiation retrieved directly from the sensor. Fipar is the fraction of photosynthetic active radiation, which was substituted by observed canopy coverage in this study. Aipar is accumulated intercepted photosynthetic active radiation, with *t* as the growth period under consideration.

### 2.4 Statistical analysis

The coefficient of determination (*R*^2^) was utilized to assess the general fit of the regression equation. Moreover, root mean square error (*RMSE*) indicates the performance of regression models. They were calculated as follows:


(7) $$R^{2}=\;\frac{\sum_{i=1}^{N}{\left(P_{i}-O_{i}\right)}^{2}}{\sum_{i=1}^{N}{\left(P_{i}-\bar{P}\right)}^{2}},$$

(8) $$RMSE=\;\sqrt{\frac{1}{N}\times \sum_{i=1}^{N}{\left(P_{i}-O_{i}\right)}^{2}},$$

where N is the number of observations, $P_{i}\;$ is the observed value, $\bar{P}$ is the mean of observed values, and $O_{i}$ is the predicted value.

The Tukey–Kramer method was applied to test the significant differences of *PNC*, DM, and *RUE* under different N treatments. The nominal alpha value of 0.05 was applied to determine *R*^2^ and significance levels. In this study, OriginPro 2023 (OriginLab Corp., Northampton, MA, USA) was used to draw all graphs, and SPSS 14.0 software (SPSS Inc., Chicago, IL, USA) was used to perform statistical analysis.

## 3 Results

### 3.1 Effect of N rates on plant N concentration and dry matter

**Table 2** displays the variation in PNC at different growth stages. In 2021, the PNC ranged from 3.02% to 5.71%, while in 2022, it ranged from 3.14% to 4.99%. In both seasons, the *PNC* of Choy Sum declined with the progress of crop growth. In 2021, the *PNC* of N_0_ and N_25_ treatments was significantly lower than that of the other treatments (N_100_, N_150_, and N_200_). The results in 2022 showed a similar pattern to 2021, although *PNC* of N_25_ only showed a significantly lower value until 25 days after transplanting.

**Table 2:** The variation in plant N concentration (*PNC*, %) of Choy Sum under different N rates in 2021 and 2022.

Year	N rates (kg/ha)	Days after transplanting				
		14	20	23	26	33
2021	0	3.50 ± 0.11 c	3.57 ± 0.30 b	3.80 ± 0.12 b	3.02 ± 0.17 b	3.12 ± 0.35 b
	25	4.75 ± 0.05 b	4.50 ± 1.28 ab	3.92 ± 0.10 b	3.76 ± 0.88 ab	3.27 ± 0.42 b
	50	5.71 ± 0.12 a	5.12 ± 0.56 a	5.30 ± 0.17 a	3.99 ± 0.62 ab	4.08 ± 0.79 ab
	100	5.58 ± 0.09 a	4.97 ± 0.75 a	5.46 ± 0.36 a	4.79 ± 0.91 a	4.43 ± 0.29 ab
	150	5.51 ± 0.12 a	5.15 ± 0.37 a	5.23 ± 0.33 a	4.67 ± 0.37 a	4.86 ± 0.74 a
	200	5.70 ± 0.20 a	4.39 ± 0.74 a	5.64 ± 0.39 a	4.44 ± 1.02 a	5.31 ± 0.33 a
Year	N rates (kg/ha)	Days after transplanting				
		16	20	–	25	–
2022	0	3.97 ± 0.47 b	4.22 ± 0.09 b	–	3.70 ± 0.71 a	–
	25	4.94 ± 0.19 a	4.68 ± 0.28 ab	–	3.27 ± 0.21 b	–
	50	4.77 ± 0.06 a	4.52 ± 0.09 ab	–	3.14 ± 0.35 b	–
	100	4.99 ± 0.09 a	4.93 ± 0.22 a	–	3.43 ± 0.37 a	–
	150	4.84 ± 0.16 a	3.55 ± 0.45 c	–	3.57 ± 0.14 a	–
	200	5.11 ± 0.20 a	4.32 ± 0.23 ab	–	3.70 ± 0.49 a	–

Values with a distinct letter after them are significant at the 5% level using Tukey–Kramer method.

The dry matter of Choy Sum varied from 4.30 g/m^2^ to 93.12 g/m^2^ in 2021 and varied from 3.87 g/m^2^ to 20.57 g/m^2^ in 2022 (**Table 3**). In 2021, a notable difference was detected in the dry matter between low N treatments (N_0_ and N_25_) and high N treatments (N_50_, N_100_, N_150_, and N_200_) at all growth stages. In 2022, there was also an obvious difference in the amount of dry matter between high N treatments (N_100_, N_150_, and N_200_) and low N treatments (N_0_ and N_25_) at 16, 20, and 25 days after transplanting.

**Table 3:** The variation in plant dry matter (g/m^2^) of Choy Sum under different N rates in 2021 and 2022.

Year	N rates (kg/ha)	Days after transplanting				
		14	20	23	26	33
2021	0	4.30 ± 0.96 b	11.79 ± 3.51 b	14.60 ± 3.02 b	19.15 ± 1.78 c	28.59 ± 1.75 c
	25	6.30 ± 0.79 ab	19.96 ± 2.21 ab	21.86 ± 2.81 b	38.80 ± 6.10 b	45.01 ± 13.28 bc
	50	7.33 ± 0.90 ab	30.24 ± 8.71 a	36.33 ± 5.71 a	52.36 ± 8.81 ab	65.90 ± 18.81 abc
	100	8.00 ± 1.31 a	33.32 ± 3.66 a	38.88 ± 5.01 a	66.14 ± 6.86 a	68.97 ± 8.22 ab
	150	6.80 ± 1.74 ab	26.76 ± 8.06 ab	40.75 ± 3.91 a	53.44 ± 11.36 ab	74.67 ± 17.33 ab
	200	7.03 ± 0.50 ab	33.45 ± 5.63 a	39.01 ± 7.26 a	59.03 ± 3.08 a	93.12 ± 15.74 a
Year	N rates (kg/ha)	Days after transplanting				
		16	20	–	25	–
2022	0	3.87 ± 0.23 d	5.70 ± 0.95 c	–	7.57 ± 0.40 b	–
	25	6.47 ± 2.02 bc	9.30 ± 2.11 bc	–	14.63 ± 2.11 ab	–
	50	5.67 ± 0.98 c	10.90 ± 2.86 ab	–	16.26 ± 2.21 ab	–
	100	6.57 ± 0.25 bc	10.73 ± 2.14 ab	–	19.27 ± 4.48 a	–
	150	7.63 ± 1.82 ab	11.80 ± 3.50 ab	–	16.90 ± 1.54 a	–
	200	8.00 ± 2.31 a	13.10 ± 2.65 a	–	20.23 ± 7.95 a	–

Values with a distinct letter after them are significant at the 5% level using Tukey–Kramer method.

### 3.2 Estimation of dry matter using vegetation indices and *Aipar*

**Figure 1:**
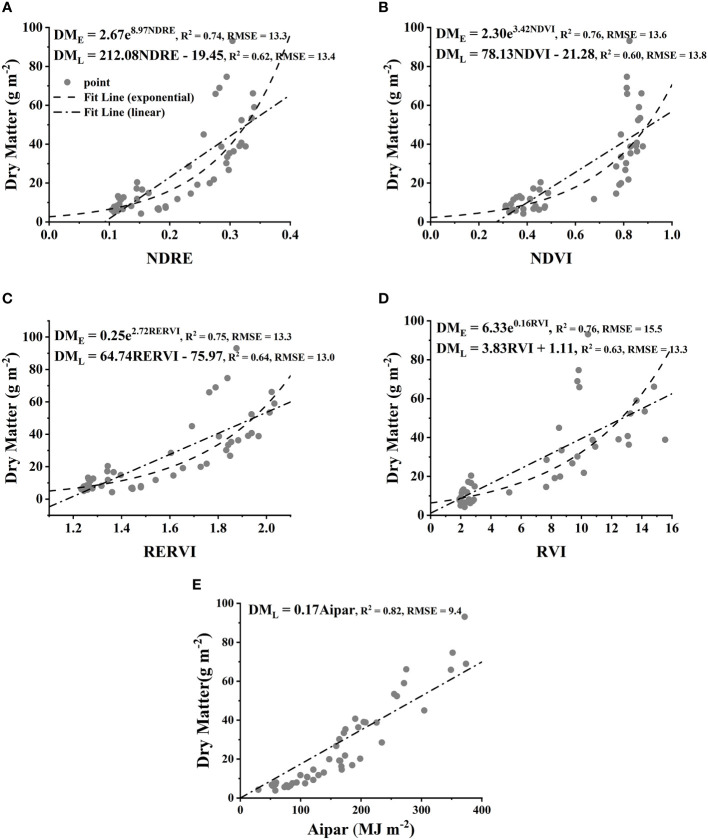
The relation between dry matter (g/m^2^) and NDRE **(A)**, NDVI **(B)**, RERVI **(C)**, RVI **(D)**, and *Aipar* **(E)** across 2021 and 2022 seasons. The number of samples is 48, and each value is the mean of three replicates. E and L stand for exponential (E) and linear (L) models, respectively. All fitted relationship functions were tested to be highly significant. NDRE, normalized difference red edge; NDVI, normalized difference vegetation index; RERVI, red edge ratio vegetation index; RVI, ratio vegetation index.

Dry matter was estimated by two different models in this study, i.e., spectral vegetation index-based and *Aipar*-based models. Dry matter and vegetation indices were observed to be closely related. Their relationship can be best fitted by exponential models (**Figure 1**), while the performances of linear models were relatively worse than those of the exponential ones. The coefficient of determination of all exponential models had considerable *R*^2^ of 0.74, 0.76, 0.75, and 0.76 for NDRE, NDVI, RERVI, and RVI, respectively. In contrast, the correlation between dry matter and *Aipar* was significantly linear (*R*^2^ = 0.82).

### 3.3 The relationship between dry matter and accumulated intercepted photosynthetic active radiation

**Figure 2:**
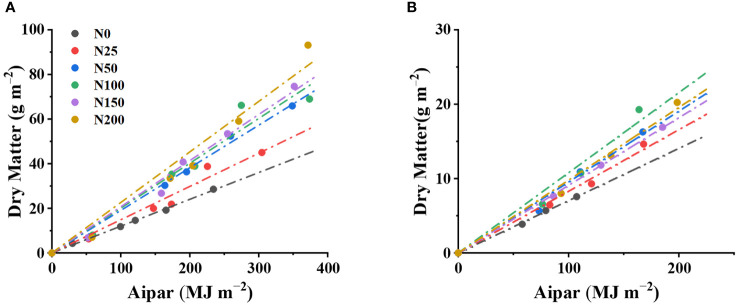
The linear correlation between dry matter and accumulated intercepted photosynthetic active radiation (*Aipar*) across different N levels in 2021 **(A)** and 2022 **(B)**.

A linear relationship between DM and *Aipar* was detected across different N rates in both 2021 and 2022 (**Figure 2**; **Table 4**). The linear relationship was derived from the equation of DM = *RUE* × *Aipar*, where *RUE* was treated as the slope of the linear function, and a comparison of *RUE* of different N treatments was also conducted. In both 2021 and 2022, the *RUE* of N_0_ and N_250_ was substantially lower than the *RUE* of other treatments. N_200_ obtained the highest *RUE* of 0.21 in 2021, while N_100_ obtained the highest *RUE* of 0.11 in 2022. Significant differences in *RUE* between N_100_, N_150_, and N_200_ were not detected. The *R*^2^ values of all regression models were higher than 0.95.

**Table 4:** Radiation use efficiency (*RUE*) of Choy Sum in 2021 and 2022.

Year	N rates (kg/ha)	*RUE*
2021	0	0.12 d
	25	0.14 c
	50	0.19 b
	100	0.20 ab
	150	0.20 ab
	200	0.21 a
2022	0	0.07 b
	25	0.08 ab
	50	0.10 a
	100	0.11 a
	150	0.09 a
	200	0.10 a

For a specific year, values with a distinct letter after them are significant at the 5% level using Tukey–Kramer method.

## 4 Discussion

### 4.1 Estimation of dry matter by spectral- and *Aipar*-based models

In this study, both *Aipar*-based and spectral reflectance-based models were adopted to estimate the dry matter yield of Choy Sum, and the performances of these two types of models were comprehensively compared. Four commonly used vegetation indices were extracted from the active field spectrometer to build estimation models of Choy Sum yield.

The relationships between vegetation indices and dry matter yield were best fitted by exponential models, which outperformed the linear ones, suggesting saturation problems occurred when dry matter approached a certain high level (**Figure 1**). This phenomenon agreed with the findings of other studies, where saturation often occurred for high biomass or LAI values in the late growth stage. For instance, it occurs when biomass reached 3 t/ha or NDVI approached 0.95 for rice (Gnyp et al., 2014), or NDVI reached 0.8 for wheat (Erdle et al., 2011), while it occurred when NDVI was approximately 0.8 for Choy Sum in this study. Nonetheless, dry matter can still be satisfactorily estimated by vegetation index-based models over the two seasons (**Figure 1**).

The saturation issue could be well addressed using the *Aipar*-based model, as *Aipar* increased proportionally to dry matter, even when the canopy approached a high level (**Figure 1**); this is due to the fact that the parameter of *Aipar* integrates not only canopy coverage but also actual sun radiation. The constraint of the study is that *Fipar* was not directly measured; alternatively, canopy coverage was used as a substitution for *Fipar*. This could lead to a deviation of the true *RUE* values, although Haverkort reported that canopy coverage could be used as an approximation of *Fipar* on potato crops (Haverkort et al., 1991; Chakwizira et al., 2015). Many studies calculated *Fipar* from remote sensing or leaf area index since there exist close relationships and turned out to be relatively accurate (Zhou et al., 2017; Chakwizira et al., 2018; Peng et al., 2021). Nonetheless, the *Fipar* estimation method in this study has rapid access at a lower cost, despite some precision sacrificed.

Furthermore, since only 2 years’ data in one site were contained in this study, and there existed many factors of environmental variation, the specific model may not be so universal over a large scale of time and space but would be a similar trend.

### 4.2 Effect of N rates on *RUE*

In both 2021 and 2022, significantly lower *RUE* was detected in non-nitrogen (N_0_) and low-nitrogen treatments (N_25_) than in the high N treatments of N_50_, N_100_, N_150_, and N_200_, among which *RUE* showed no difference. This indicated that the accumulation of dry matter was caused by the increase of not only *Aipar* but also *RUE*. When the N rate was above 50 kg N/ha, *RUE* will not be a restriction factor for DM accumulation, implying DM increase was entirely caused by *Aipar* increase resulting from N application. Crops have different strategies to cope with N deficiency; some crops maintain chlorophyll content to keep constant radiation use efficiency, i.e., with *RUE* unaffected. The research in potatoes showed that *RUE* remained unchanged at different N levels (Millard and Marshall, 1986; Bangemann et al., 2014), as potatoes tend to reduce the leaf area instead of photosynthesis efficiency per unit leaf area when N is insufficient (Vos and van der Putten, 1998); a similar phenomenon is also demonstrated on beet crops (Chakwizira et al., 2018). In contrast, other studies showed that *RUE* increased significantly with N application and leveled off when the N rate reached to medium level on corn (Muchow and Davis, 1988; Kaur et al., 2012; Kar and Kumar, 2016). In the case of Choy Sum, increased leaf N content was mainly used for thylakoid accumulation in photosynthetic cells and synthesis of carboxylases, rather than increasing leaf area to capture light (Khairun et al., 2016), corroborating well with the discovery of this research. In conclusion, N application significantly increases the *RUE* of Choy Sum when the N rate is below 50 kg N/ha. However, beyond this threshold, further increases in the N rate did not affect *RUE*.

## 5 Conclusions

This study evaluated the performance of *Aipar* and spectral-based methods for estimating the dry matter yield of Choy Sum over two growing seasons. The results showed that the relationships between DM of Choy Sum and vegetation indices were best fitted by exponential models due to the saturation problems in the case of high biomass, while *Aipar* had a significant linear relationship with DM (*R*^2^ = 0.82) even when the canopy approached a high level. This suggests that *Aipar* could be used as a better candidate for dry matter yield estimation. With regard to the effect of N application on the *RUE* of Choy Sum, the *RUE* of crops applied with low N (0–25 kg N/ha) was significantly lower than crops applied with high N (50–200 kg N/ha). Therefore, when applied with a N rate above 50 kg N/ha, dry matter yield production of Choy Sum will not be constrained by reduced *RUE*.

## Data availability statement

The raw data supporting the conclusions of this article will be made available by the authors, without undue reservation.

## Author contributions

Conceptualization, ZW, YH, and ZZ; methodology, ZW; software, ZW and JP; formal analysis, SS and LZ; investigation, JS, SS, SC, DQ, and ZZ; data curation, ZZ; writing—original draft, ZW and YH; writing—review and editing, SC, JS, YL, XW, and ZZ; supervision, ZZ; funding acquisition, ZZ. All authors contributed to the article and approved the submitted version.
